# Exploring the Therapeutic Potential of Benfotiamine in a Sporadic Alzheimer’s-Like Disease Rat Model: Insights into Insulin Signaling and Cognitive function

**DOI:** 10.1021/acschemneuro.4c00113

**Published:** 2024-07-15

**Authors:** Camila A. E. F. Cardinali, Yandara A. Martins, Ruan C. M. Moraes, Andressa P. Costa, Mayke B. Alencar, Ariel M. Silber, Andrea S. Torrão

**Affiliations:** † Departamento de Fisiologia e Biofisica, Instituto de Ciencias Biomedicas, Universidade de Sao Paulo, Sao Paulo 05508-000, Brazil; ‡ Department of Psychiatry & Behavioral Neurosciences, The University of Alabama at Birmingham, Birmingham Alabama 35294, United States; § Laboratory of Biochemistry of Tryps−LaBTryps, Departamento de Parasitologia, Instituto de Ciencias Biomedicas, Universidade de Sao Paulo, Sao Paulo 05508-000, Brazil

**Keywords:** neurodegeneration, cognitive decline, thiamine, vitamin B1, neuroprotection, streptozotocin

## Abstract

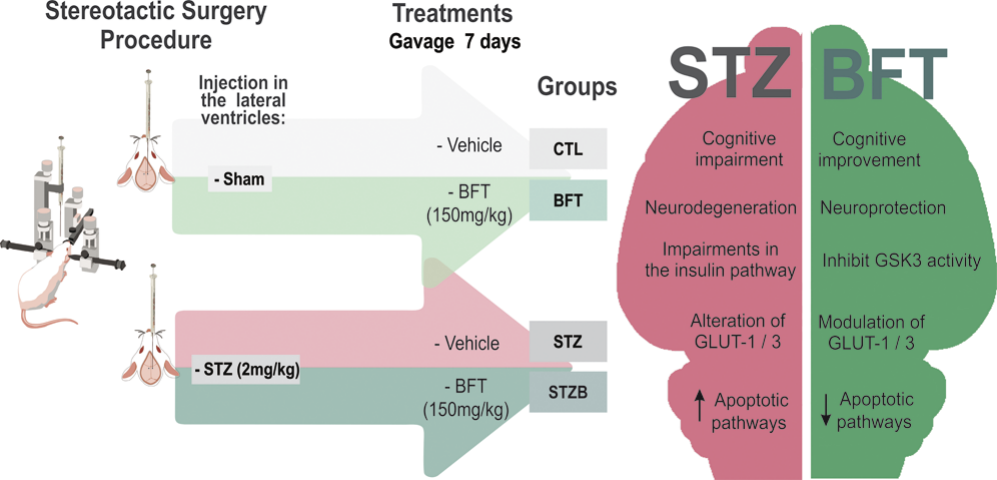

Alzheimer’s disease (AD) is a complex neurodegenerative process, also considered a metabolic condition due to alterations in glucose metabolism and insulin signaling pathways in the brain, which share similarities with diabetes. This study aimed to investigate the therapeutic effects of benfotiamine (BFT), a vitamin B1 analog, in the early stages of the neurodegenerative process in a sporadic model of Alzheimer’s-like disease induced by intracerebroventricular injection of streptozotocin (STZ). Supplementation with 150 mg/kg of BFT for 7 days reversed the cognitive impairment in short- and long-term memories caused by STZ in rodents. We attribute these effects to BFT’s ability to modulate glucose transporters type 1 and 3 (GLUT1 and GLUT3) in the hippocampus, inhibit GSK3 activity in the hippocampus, and modulate the insulin signaling in the hippocampus and entorhinal cortex, as well as reduce the activation of apoptotic pathways (BAX) in the hippocampus. Therefore, BFT emerges as a promising and accessible intervention in the initial treatment of conditions similar to AD.

## Introduction

Alzheimer’s disease (AD), a prevalent neurodegenerative disease, impacts millions and is projected to cost $2 billion annually by 2030.^1^ Ninety-five percent of AD cases are sporadic (SAD) with aging being the main risk factor.^2^ β-Amyloid (Aβ) protein plaque accumulation, neurofibrillary tangles (NFT), and neuroinflammation are the hallmarks of AD.^3,4^ So far, however, clinical efforts to improve cognitive function through modifications of Aβ and Tau were unsuccessful.^5,6^ Recent mass spectrometry-based proteomics reinforced the heterogeneity of AD, categorizing it into 5 different forms.^7^ Despite all advances in understanding AD, the etiology of the disease remains unclear. Thus, it is necessary to recognize the complexity of AD and the need for additional research to comprehend the mechanisms of the disease and to develop efficient treatments.

Quantitative proteomics of more than 2,000 brains and 400 cerebrospinal fluid samples identified therapeutic targets and biomarkers for AD, which included glucose metabolism, mitochondrial changes, and neuroinflammation.^8^ Studies show convincing evidence that AD is a metabolic disease (changes in glucose metabolism and in insulin signaling pathway).^9−12^ This hypothesis was based on similar traits observed in AD and diabetic patients.^11,13−17^

Insulin plays neurotrophic, neuromodulatory, and neuroendocrine roles in the brain.^11,18^ The insulin signaling pathway regulates neuronal apoptosis, β- and γ-secretases activity, amyloid precursor protein (APP), and Tau phosphorylation.^19−21^ Alterations in its pathway can lead to Aβ buildup, NFT formation, oxidative stress, neuroinflammation (activation of astrocytes and microglia), changes in glucose metabolism and apoptosis, elements present in AD.^11−13,16,18^

Thiamine (B1 vitamin) has a central role in the energetic metabolism of the brain.^22^ The decline in thiamine-dependent enzyme activity impacts the supply of substrates for neurotransmitter synthesis (e.g., glutamate, acetylcholine, GABA), mitochondrial activity, oxidative stress, neuroinflammation, calcium metabolism, and cognitive function.^23−26^ Despite being essential, the free thiamine transport rate is saturable. Thus, membrane transport is a limiting step of the treatment. To compensate for this issue, thiamine analogs with greater bioavailability were developed.^19^

Benfotiamine (BFT) increases the bioavailability of thiamine nearly five times.^22^ It was initially used to prevent complications in diabetes models.^27^ Subsequent research demonstrated the neuroprotective effects of BFT in a model of neurodegeneration.^19,22,28,29^ The mechanisms of action of BFT remain unclear, especially those promoted by the diphosphate form of thiamine (ThDP).

Therefore, the aim of the present work was to assess the short-term effects of BFT on behavior, biochemical and molecular parameters related to insulin signaling, glucose transporters, mitochondrial activity, and neurodegeneration mechanisms in a nontransgenic metabolic model of SAD induced by intracerebroventricular (icv) injection of streptozotocin (STZ) in rats. Icv-STZ promotes brain alterations and cognitive deficits similar to those encountered in SAD. Thus, STZ-icv is commonly used as a nontransgenic metabolic model for SAD.^30,31^

## Results and Discussion

Our results demonstrate that 7-day BFT treatment reverses the cognitive impairment for short- and long-term memories promoted by STZ. STZB animals also had decreased anxiety-like behavior. We believe that these effects are a consequence of the BFT-linked modulation of pathways involved with synaptic plasticity, memory, cell growth and proliferation.

### STZ Has an Anxiolytic Effect on Animal Behavior

**Figure 1:**
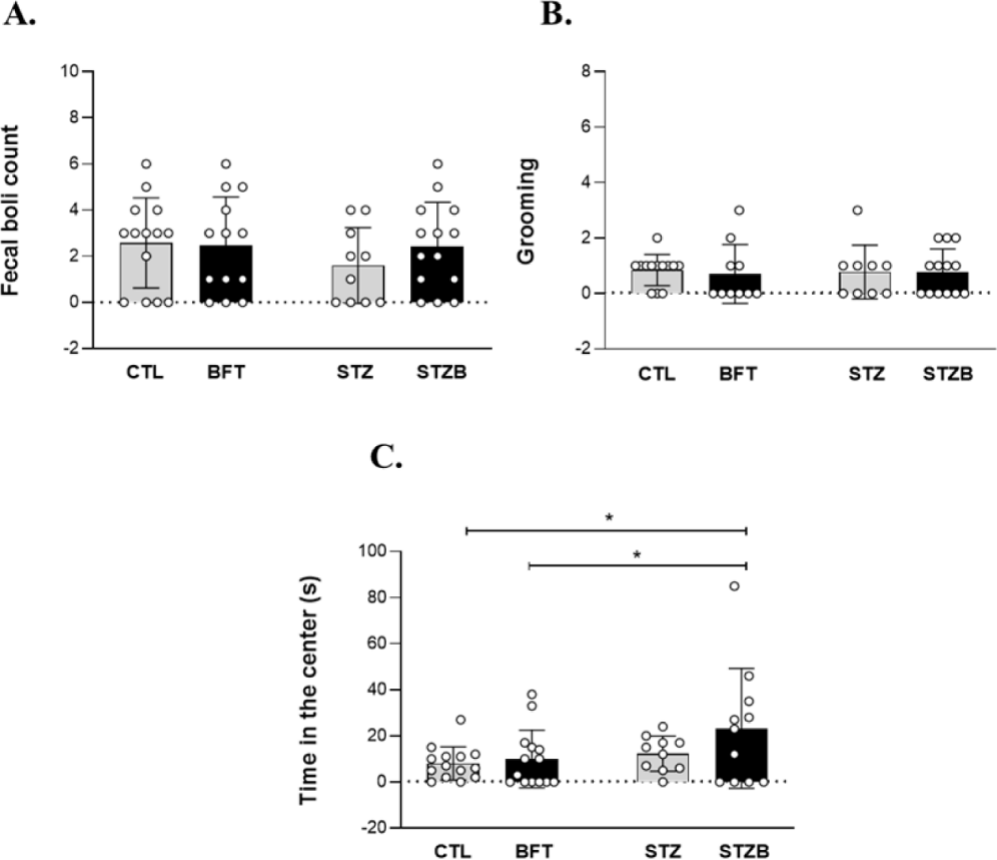
Effect of 7-day-BFT treatment in the anxiety-like behavior of rats in 5 min of the open field test (OF). (A) Fecal boli count. (B) Groomings. (C) Time in the center (s). Results are presented as mean ± SD (*n* = 9–14).*p < 0.05.

The OF test was used to evaluate motor activity and exploratory and anxious-like behaviors of rodents. Zone crossings and rearing did not differ between groups (Figure S1), suggesting unaltered motor function and exploratory behavior, which could bias our results in the following behavior tests. OF and EPM were used to evaluate the anxious-like behavior of rodents. The basis of these tests is the conflict between the animal’s impulse and curiosity in exploring the unknown and motivation to avoid dangerous environments. In the OF, there were no differences in fecal boli count (Figure 1A) and grooming (Figure 1B). STZB group spent 187.48% (F(1.45) = 1.099, *p* = 0.015) and 132.5% (F(1.45) = 1.099, *p* = 0.032) more time in the central area of the arena in comparison to CTL and BFT, respectively (Figure 1C). This suggests a decreased anxiety-like behavior in the STZB group. Rodents tend to avoid the central area of the arena and prefer to circulate near its walls (thigmotaxis), and increased time spent in the center indicates less anxiety.

**Figure 2:**
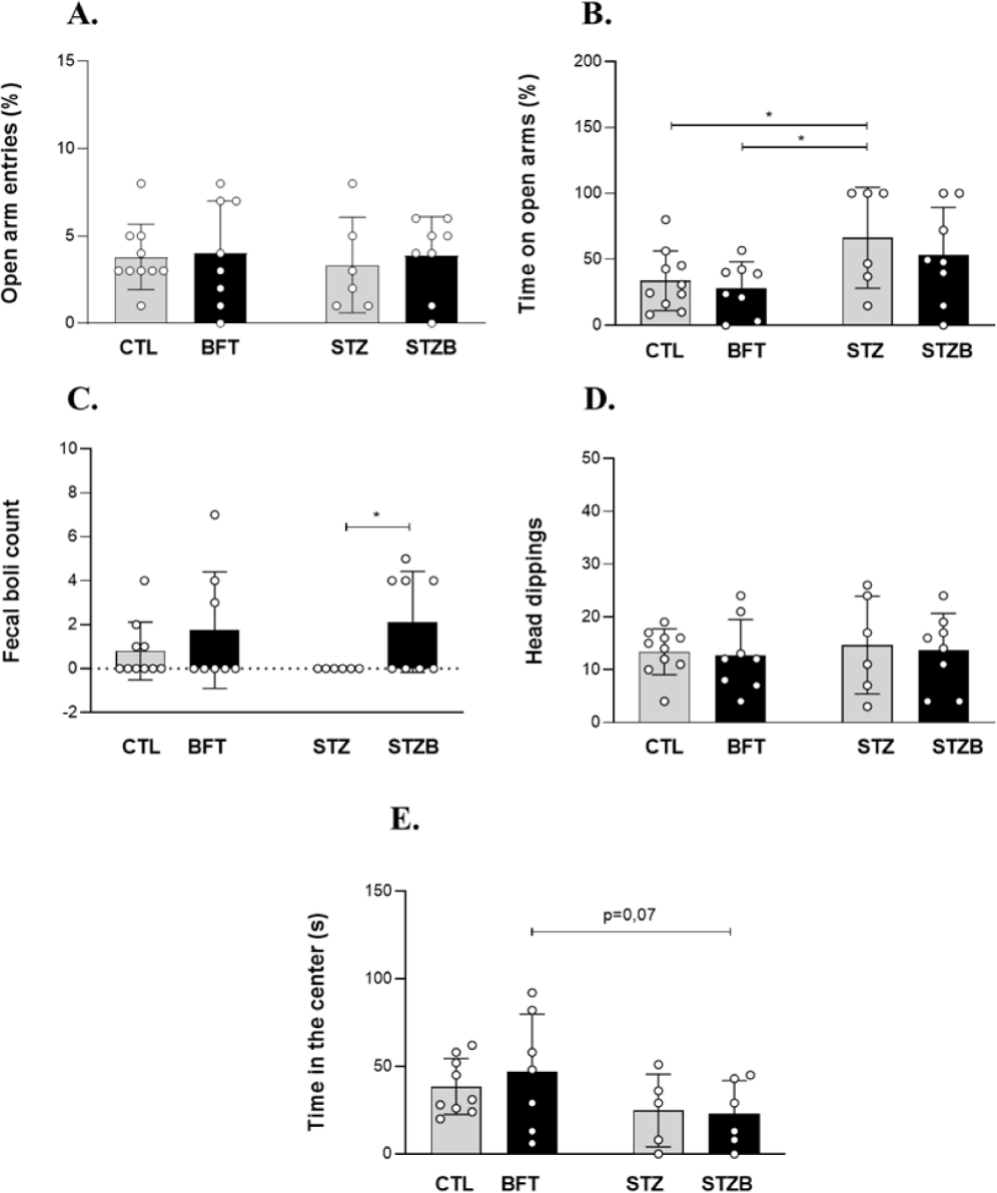
Effect of 7 day-BFT treatment in the anxiety-like behavior of rat in 5 min of the elevated plus maze. (A) Open arm entries (%). (B) Time on open arms (%). (C) Fecal boli count. (D) Head dippings. (E) Time in the center (s). Results are presented as mean ± SD (*n* = 5–10). *p < 0.05.

The increased time spent in the open arms vs total time in the EPM indicates lower anxiety levels. We did not observe differences between groups in open-arm entries (Figure 2A). Nonetheless, STZ group spent more time in the open arms of the maze in comparison to BFT, an increase of 135.46% (F(1.28) = 0.1425, *p* = 0.039) and 87.87% (F(1.28) = 0.1425, *p* = 0.022) in comparison to CTL, respectively (Figure 2B). The increased exploration time of the open arms (danger zone) indicates a decrease in anxious-like behavior and can also be associated with impulsivity.^32,33^ Rodents exposed to a new/risky environment have an increase in fecal boli due to the activation of the autonomous nervous system, positively correlated to emotionality. BFT treatment increased fecal boli count for the STZB group compared to STZ (F(1.28) = 0.7337, *p* = 0.038, Figure 2C), suggesting a greater emotionality for this group.

Time spent in the central platform is associated with decision-making and risk assessment, representing a conflict response between dodge/approach in the open arm.^34−36^ In addition to spending less time in unprotected areas, BFT animals stayed more time in the central zone of the maze, suggesting a more controlled and less impulsive behavior compared to STZB (F(1.23) = 0.3203, *p* = 0.07, Figure 2E**)**. During the EPM, three STZ and three STZB animals jumped from the maze and were excluded from the analysis. Our results show that the STZ groups are less anxious, have deficits in unconditioned fear, are more impulsive, and have a compromised risk assessment.

STZ-icv is an SAD model. It shares similarities to cognitive, morphological and behavioral alterations (e.g., hyperactivity, increased exploratory behavior and lower anxiety) encountered in AD patients.^30,31,37−41^ Impairments in connections between the hippocampus, entorhinal cortex, and amygdala promote decreased unconditioned fear and loss of risk assessment. Lesions in the ventral area of the hippocampus are also related to a lesser anxious-like behavior.^42,43^ Our previous work revealed working memory impairments 3 h after the STZ-icv injection and a degenerative process in rat hippocampus that lasts for up to 15 days, specifically in the CA1 area.^44^ Therefore, the changes in behavior we observed could be related to STZ-induced hippocampal lesions.

### Benfotiamine Reverts the STZ-Induced Cognitive Impairment

**Figure 3:**
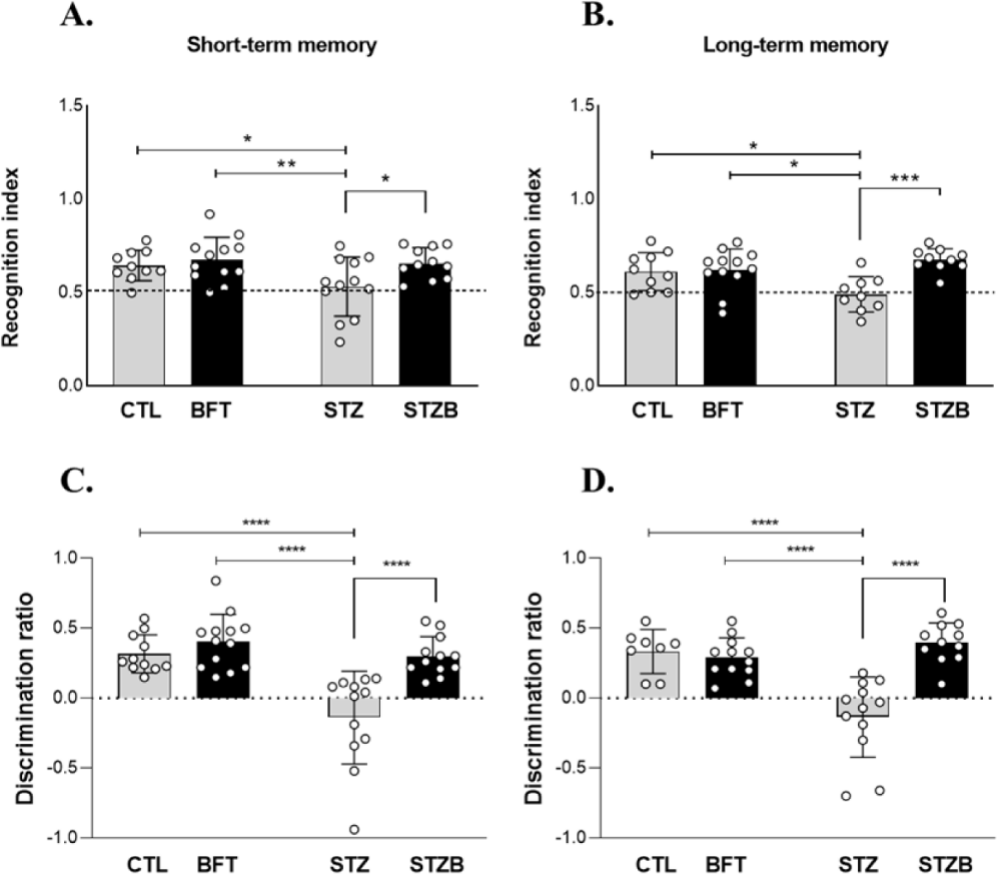
Novel object recognition test. Recognition index (RI) for (A) short-term memory (STM) and (B) long-term memory (LTM). Discrimination ratio (DR) for (C) STM and (D) LTM. Results are presented as mean ± SD (*n* = 8–13). *p < 0.05; **p < 0.01; ***p < 0.001.

To verify if the increase in BFT metabolites impacted cognitive function, we evaluated short- (STM) and long-term memories (LTM) through the novel object recognition test. For both memories evaluated, the STZ group showed cognitive impairment with a reduction in recognition index (RI) of 17.18% (F(1.41) = 1.862, *p* = 0.02) and 19.67% (F(1.37) = 8.730, *p* = 0.008) compared to CTL for STM (Figure 3A) and LTM (Figure 3B**)**, respectively. Discrimination ratio (DR) is an analog of the recognition memory sensitivity. STZ animals showed a decrease of 145% (F(1.44) = 7.896, *p* < 0.001) and 140% (F(1.39) = 22.400, *p* < 0.001) in DR compared to CTL in STM and LTM, respectively (Figure 3C,D). These results indicate that STZ animals cannot discriminate between familiar and novel objects. The 7-day BFT treatment reverted the cognitive deficit in both tests.

### STZ Increases GluN2b Levels, which Is Attenuated by BFT Supplementation

**Figure 4:**
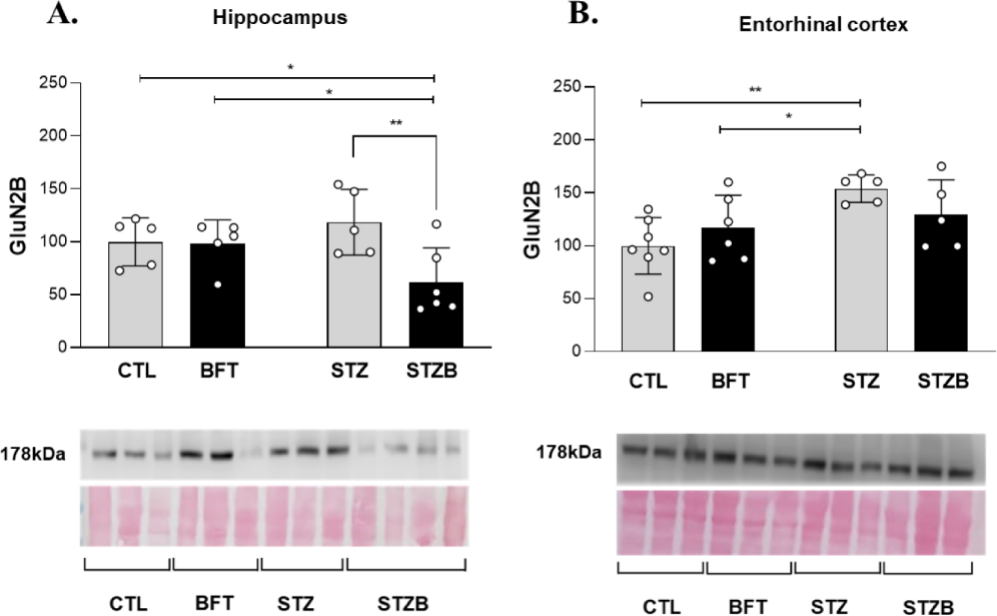
Effect of 7-day-BFT treatment in the levels of GluN2B detected by immunoblotting in the (A) hippocampus and (B) entorhinal cortex. Results are presented as mean ± SD (*n* = 5–7) *p < 0.05; **p < 0.01.

The GluN2B subunit of the NMDA glutamate receptor is required for long-term potentiation (LTP) and memory. However, its activation has also been linked with AD progression when it promotes excessive calcium influx, leading to excitotoxicity. To evaluate if GluN2B alterations could be behind the cognitive impairment seen in the STZ group and its reversal with BFT treatment, we assessed GluN2B levels. BFT treatment reduced by 56.47% (F(1.17) = 5.054, *p* = 0.003) the levels of GluN2B in the hippocampus of the STZB group compared to STZ (Figure 4A). In the entorhinal cortex, GluN2B levels rose by 54.03% (F(1.19) = 3.340, *p* = 0.037) and 36.81% (F(1.19) = 3.340, *p* = 0.045) of the STZ group compared to CTL and BFT groups, respectively (Figure 4B).

STZ-induced cognitive impairment for short- and long-term memories was reverted with BFT treatment. Glutamatergic neurotransmission via NMDA receptors is crucial for synaptic plasticity and neuronal survival.^45^ The overactivation of its GluN2B subunit leads to excessive calcium influx and excitotoxicity.^46^ Memantine is used to attenuate AD symptoms, supporting the involvement of NMDA receptors in the onset and course of the disease.^45^ Despite not considering the phosphorylation and activation, we found increased levels of GluN2B, after icv-STZ. Curiously, this effect was prevented by BFT treatment in the STZB group. Liu et al. (2023) showed that STZ decreases the levels and phosphorylation of GluN2B, indicating a dysregulation in cell membrane trafficking of GluN2B after its overactivation.^46^ In the same model used here, Moraes et al. (2020) observed a reduction in GluN2B density in STZ animals and BFT-30-day treatment restored these levels. We hypothesized that in the short term, STZ induces GluN2B expression, resulting in hyperexcitability. However, 30-day BFT treatment reduces the expression of GluN2B which could control the hyperexcitability. Therefore, BFT may prevent hyperexcitability,^28^ explaining the lack of short-term increase and long-term decrease in GluN2B expression.

### BDNF Expression Increases in the Hippocampus of STZB and in the Entorhinal Cortex of STZ Groups

**Figure 5:**
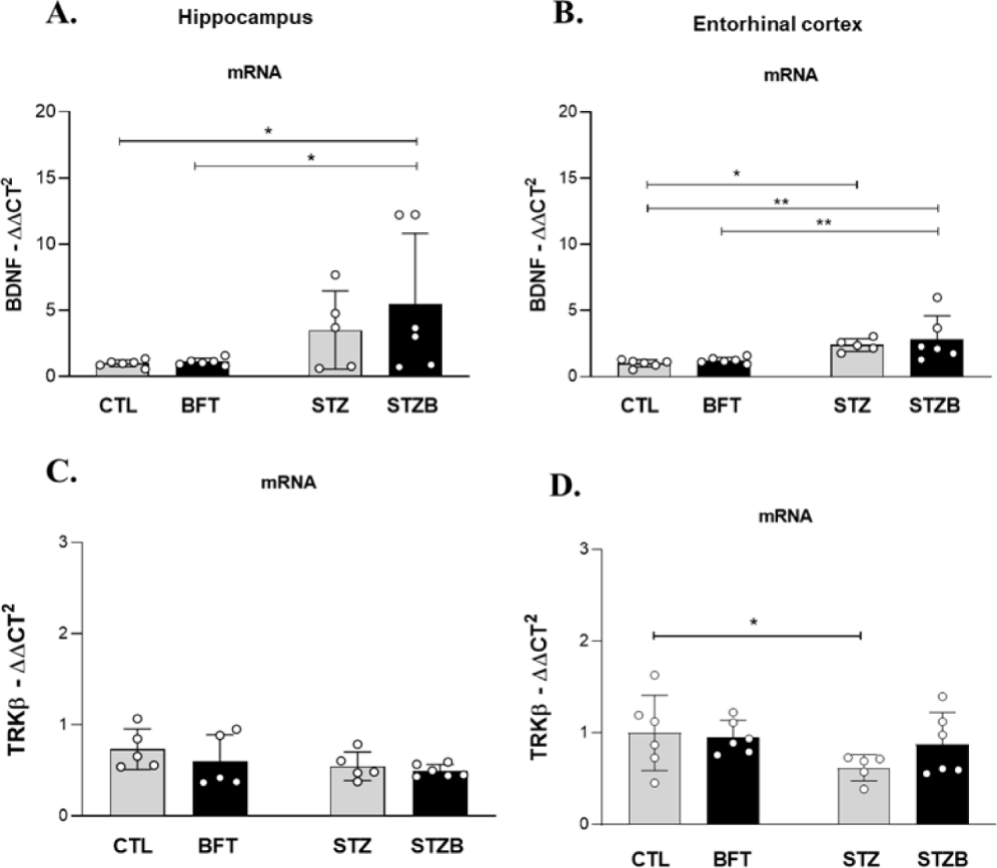
Effect of 7-day-BFT treatment in mRNA levels of brain-derived neurotrophic factor (BDNF) and TRKβ receptors in the (A and C) hippocampus and (B and D) entorhinal cortex. Results are presented as mean ± SD (*n* = 5–6). *p < 0.05; **p < 0.01.

The brain-derived neurotrophic factor (BDNF) has a role in the growth and maintenance of neurons, synaptic plasticity, and cognitive function. Recent studies suggest that BDNF and its specific tyrosine kinase receptor (Trkβ) also regulate energetic homeostasis.^47^ Treatments capable of improving the energetic response, such as BFT, can modulate this pathway and promote a neuroprotective effect. We observed an increase in BDNF expression in the STZB group in the hippocampus and entorhinal cortex (Figure 5). This increase was 440% (F(1.19) = 0.504, *p* = 0.02) and 180% (F(1.19) = 0.080, *p* = 0.002) higher for STZB compared to CTL animals in the hippocampus and entorhinal cortex, respectively. Compared to BFT, the STZB group had an increase of 350% (F(1.19) = 0.504, *p* = 0.02) and 133% (F(1.19) = 0.080, *p* = 0.007) in the hippocampus and entorhinal cortex, respectively. In the entorhinal cortex, there was also an increase in BDNF expression for STZ animals (130%, F(1.19) = 0.080, *p* = 0.02) compared to CTL. Trkβ receptor hippocampal expression was similar between groups (Figure 5C). In the entorhinal cortex, the expression of Trkβ decreased by 38.02% (F(1.19) = 1.490, *p* = 0.048) in the STZ group compared to CTL (Figure 5D).

BDNF has a role in neuron growth and maintenance, synaptic plasticity, cognitive function, and regulation of the energetic homeostasis.^47^ It also increases the synthesis of neuronal ATP by promoting glucose (increase in GLUT3 expression) and amino acid transport and protein synthesis.^48^ BDNF protects neurons from apoptosis by activating antiapoptotic genes, such as Bcl-2, and inhibiting pro-apoptotic proteins, like BAX and BAD.^47^ Excitatory synaptic stimuli, neuropeptides and hormones (e.g., insulin) activate brain synthesis and secretion of BDNF.^49^ Calcium ions activate sensitive enzymes (e.g., calmodulin kinase, kinase C protein, kinase enzymes activated by mitogen) that stimulate transcription factors like the cyclic AMP response-binding protein (CREB) and nuclear factor-KappaB (NF-κB), responsible for BDNF transcription.^47^

Animal studies confirm the role of a decrease in BDNF signaling in neurodegenerative disorders, such as AD.^47,49^ Furthermore, oxidative stress contributes to cell death by decreasing the activity of the TrkB/BDNF system and lowering neuronal activity.^50^ Our results demonstrate, however, that this does not occur in the initial phases of the neurodegenerative process induced by STZ, in which we observed an increase in BDNF expression. In our previous study, STZ-induced toxicity was mediated by the generation of nitric oxide (NO) and reactive oxygen species.^51^ Changes in neuron maintenance, both in structural and metabolic aspects, affect BDNF expression in STZ animals by mechanisms that still need to be enlightened. BFT treatment had no effect on BDNF expression.

### STZ and BFT Do Not Alter Mitochondrial ATP Production

**Figure 6:**
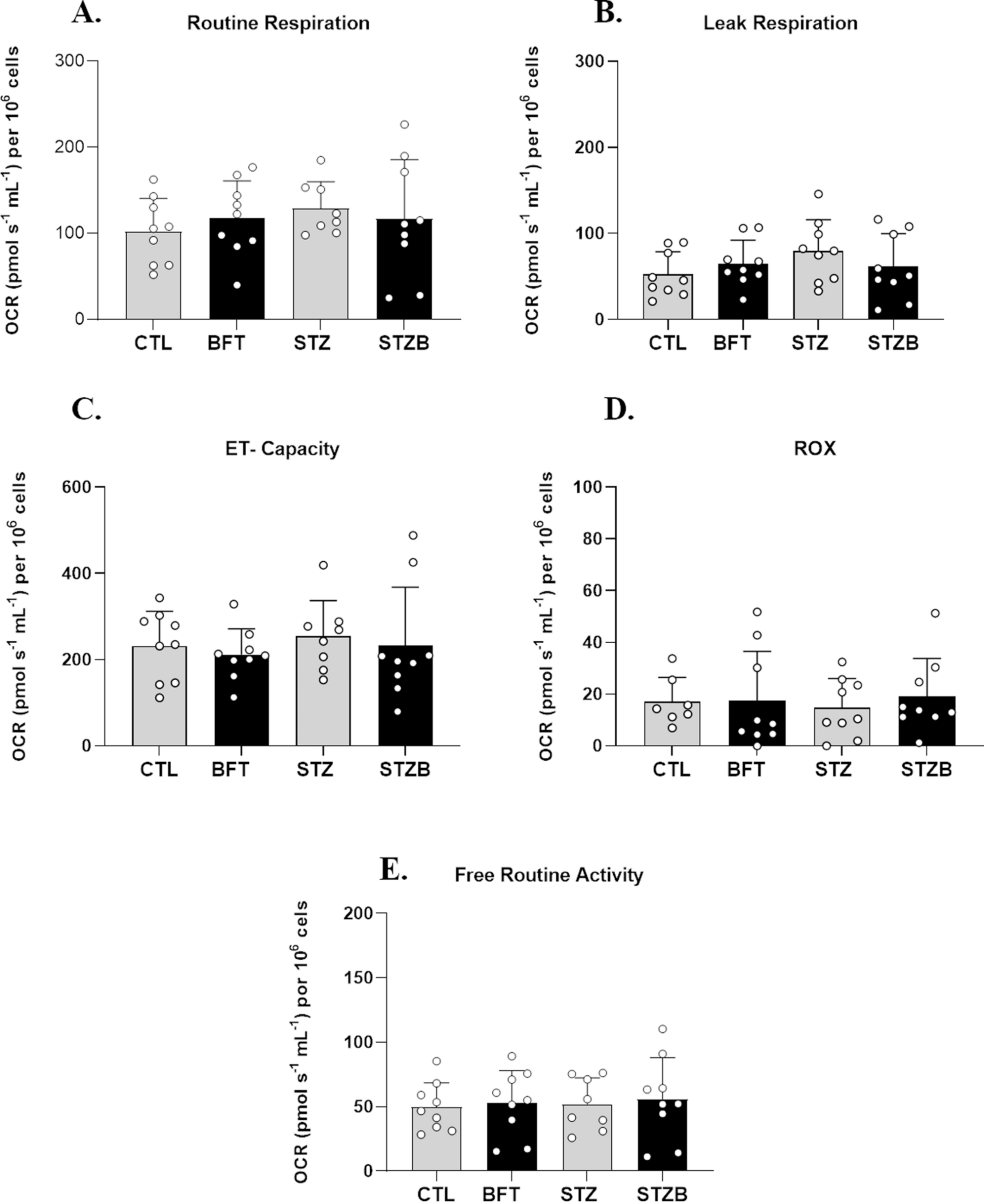
Effect of BFT (50 μM, 24h) and STZ (5 mM, 29h) treatments on mitochondrial activity of neuroblastoma 2a (Neuro2a) cell line. (A) Routine respiration. (B) Leak respiration. (C) Electron transfer (ET)—Capacity is maximum respiratory capacity after addition of FCCP and (D) ROX and respiratory rate after electron transfer inhibition by antimycin. (E) The oxygen consumption rate linked to ATP production (OCR). Results are presented as mean ± SD (*n* = 8–9). *p < 0.05.

In a previous study, we observed a decrease in mitochondrial respiration in hypothalamic GABAergic cells exposed to STZ.^28^ As BFT influences bioenergetics, given the association of thiamine with energy metabolism, our interest focused on examining the effects of STZ and BFT on a cholinergic cell line, given the importance of the cholinergic system in Alzheimer’s disease.^52^ Mitochondria plays a central role in energy metabolism and regulation of cell death, influencing ATP production, reactive oxygen species (ROS) formation, intracellular calcium homeostasis, and apoptosis.^53,54^ Impairments in oxidative phosphorylation (OXPHOS) result in decreased ATP generation, contributing to oxidative stress associated with the onset of AD.^53,54^ Considering the role of the diphosphate form of thiamine as an enzymatic cofactor in energy metabolism reactions, mitochondrial activity was evaluated in neuroblastoma 2a cells exposed to STZ and BFT (Figure 6).

Routine mitochondrial respiration did not differ between groups (Figure 6A). Oligomycin was used to block mitochondrial ATP synthesis and proton translocation and allowed us to assess the leak respiration, where we also found no changes between treatments (Figure 6B). The proton gradient was dissipated by FCCP titration until the maximum electron transfer system capacity was reached (Figure 6C). The addition of antimycin A blocked mitochondrial respiration at complex III, indicating the residual consumption of oxygen (ROX) (Figure 6D). Neither STZ nor BFT supplementation affected mitochondrial respiration. The oxygen consumption rate linked to ATP production (OCR) also showed no difference between the groups (Figure 6E).

Our in vitro results suggest no changes in mitochondrial ATP production. Nevertheless, STZ could have other mitochondrial effects, such as ROS production. The increase in ROS and inflammation are well described in this model,^38,51,55^ BFT effects may occur via improvements in oxidative stress and inflammation. BFT-30-day treatment improved the pyruvate dehydrogenase activity, indicating that beyond increasing brain levels of ThDP, it also potentiates the related metabolic activity.^28^

### STZ Increases the Levels of Thiamine Receptor and Benfotiamine Treatment Impacts Its Levels in the Hippocampus and Entorhinal Cortex

**Figure 7:**
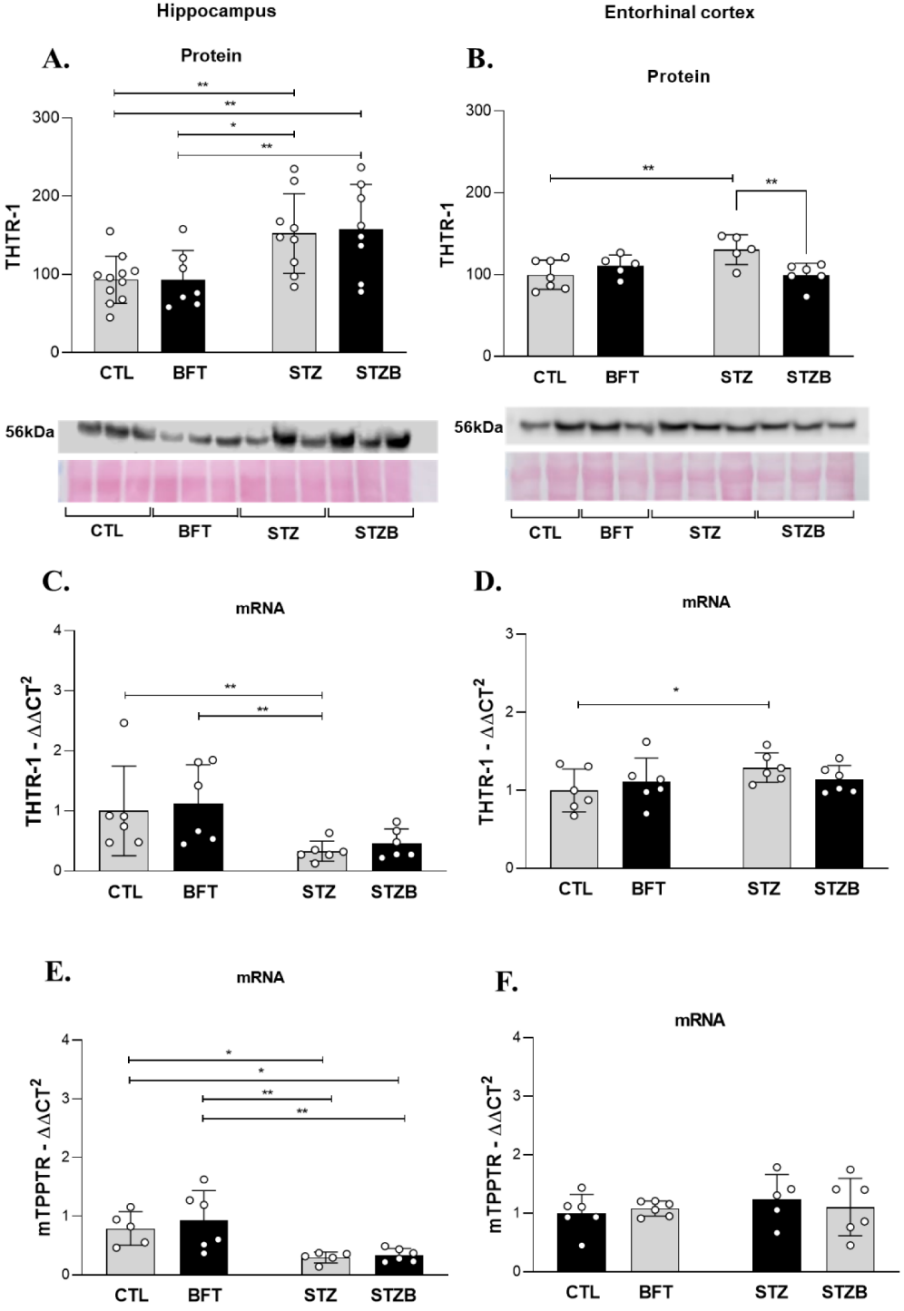
Effect of 7 day-BFT treatment in the levels of thiamine receptor (THTR-1) detected by immunoblotting in the (A) hippocampus and (B) entorhinal cortex. mRNA levels of THTR-1 in the (C) hippocampus and (D) entorhinal cortex. mRNA levels of mitochondrial THTR (mTPPTR) in the (E) hippocampus and (F) entorhinal cortex. Results are presented as mean ± SD (*n* = 5–11). *p < 0.05; **p < 0.01.

B1 vitamin (thiamine) is an essential vitamin with a main role in the energetic metabolism. BFT supplementation increases the bioavailability of thiamine. To investigate if the impairment in cell and mitochondrial thiamine transport can compromise the effects of the treatment, we evaluated the levels and gene expression of thiamine transporters THTR-1 and mTPPTR in the hippocampus and entorhinal cortex. Thiamine transporter type 1 was evaluated due to its higher expression in brain tissue. The thiamine transporter type 2 (THTR-2) was not evaluated because it is more specific to endothelial cells, probably in the blood–brain barrier. In the hippocampus, the levels of THTR-1 increased in the STZ group compared to CTL (59.22%, F(1.31) = 0.027, *p* = 0.005) and BFT (58.69%, F(1.31) = 0.027, *p* = 0.013, Figure 7A). STZB animals also showed an increase in THTR-1 compared to CTL (64.61%, F(1.31) = 0.027, *p* = 0.003) and BFT (64.28%, F(1.31) 0.027, *p* = 0.008, Figure 7A). mRNA levels of THTR-1 (H = 10.69, *p* < 0.05, Figure 7C) and mTPPTR (F(1.18) = 0.141, *p* < 0,05, Figure 7E) decreased in STZ and STZB groups compared to controls. In the entorhinal cortex, only STZ animals had an increase in THTR-1 levels (30.5%, F(1.19) = 9.290, *p* = 0.004) and expression (29.5%, F(1.20) = 1.720, *p* = 0.04) compared to CTL (Figure 7B and D). BFT treatment normalized THTR-1 levels of the STZB group (F(1.19) = 9.290, *p* = 0.005, Figure 7B).

Changes in glucose uptake and metabolism deficits also contribute to cognitive impairment. Studies have shown that brain glucose hypometabolism begins before brain atrophy and AD clinical symptoms.^9,56 −58^ Lower glucose supply to brain cells impacts the metabolism and other ATP-dependent process, such as synaptic activity and signal transmission through neurons.^47,59^ Thus, drugs capable of modulating brain energy metabolism and mitochondrial function are promising in AD treatment. BFT is an analog of the B1 vitamin (thiamine) with greater bioavailability. Despite contradictory results,^60,61^ other studies showed that BFT increases thiamine and thiamine diphosphate (ThDP),^28,62,63^ It is worth mentioning that, in our previous study,^28^ we used homogenates from specific brain regions (e.g., hippocampus and entorhinal cortex) instead of whole-brain homogenates, which could explain this discrepancy. We demonstrated that chronic BFT supplementation increases ThDP levels in hippocampus and entorhinal cortex^28^ and showed higher plasma levels of free thiamine, thiamine monophosphate and ThDP.^29^

ThDP is a cofactor of rate-limiting enzymes in the Krebs cycle and the pentose phosphate pathway.^18,64^ A compromised BFT metabolite uptake could limit the effects of the treatment. Despite STZ decreasing the gene expression of both transporters, their density increased in the hippocampus, which could be a compensatory effect (lower degradation due to the STZ-induced metabolic stress).

There is a distinct variation in responses between the regions studied, such as the hippocampus and the entorhinal cortex. The hippocampal–entorhinal system plays a crucial role in cognition and is especially susceptible to Alzheimer’s disease (AD).^65^ Although these regions are closely linked anatomically and functionally, previous studies, both those conducted by our group^28,29^ and others,^65^ have revealed striking molecular differences in their gene and protein expression profiles. Specifically in AD, recent research has identified 454 genes differentially expressed in the entorhinal cortex, with only 223 of them coinciding with those found in the hippocampus.^65^ A principal component analysis (PCA) showed considerable similarity between the CA1, CA3, and CA4 regions of the hippocampus, but no significant correspondence with CA2 and the entorhinal cortex, highlighting the distinct signature of the latter structure.^65^ Given the consistency of this disparity between the hippocampus and entorhinal cortex across different studies, it is essential to investigate them independently, without assuming uniform results between both.

### GLUT1 and GLUT3 Are Modulated by STZ and Benfotiamine in the Hippocampus

**Figure 8:**
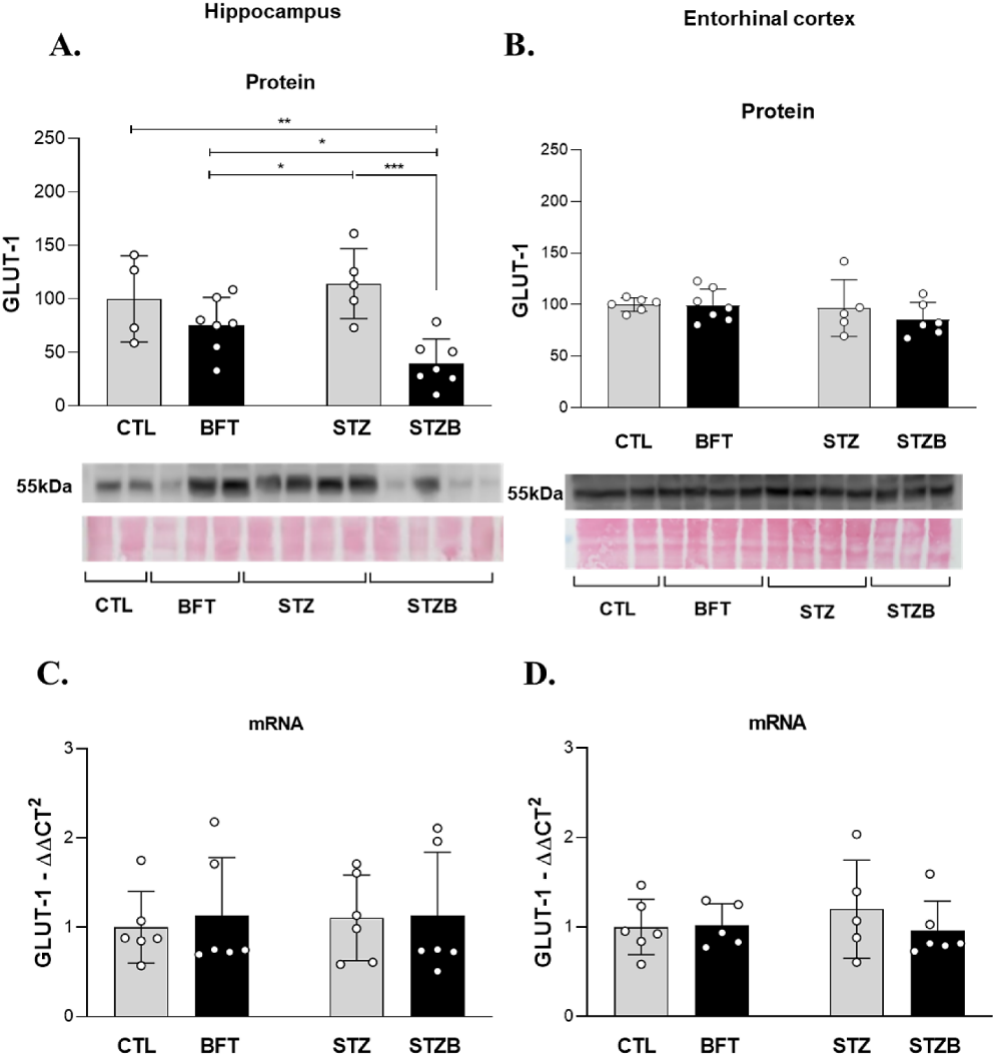
Effect of 7 day-BFT treatment in type 1 glucose transporters (GLUT1). (A) GLUT1 in the hippocampus detected by immunoblotting. (B) GLUT1 in the entorhinal cortex detected by immunoblotting. (C) mRNA levels of GLUT1 in the hippocampus. (D) mRNA levels of GLUT1 in the entorhinal cortex. Results are presented as mean ± SD (*n* = 4–7). *p < 0.05; **p < 0,0.1; **p < 0.001.

**Figure 9:**
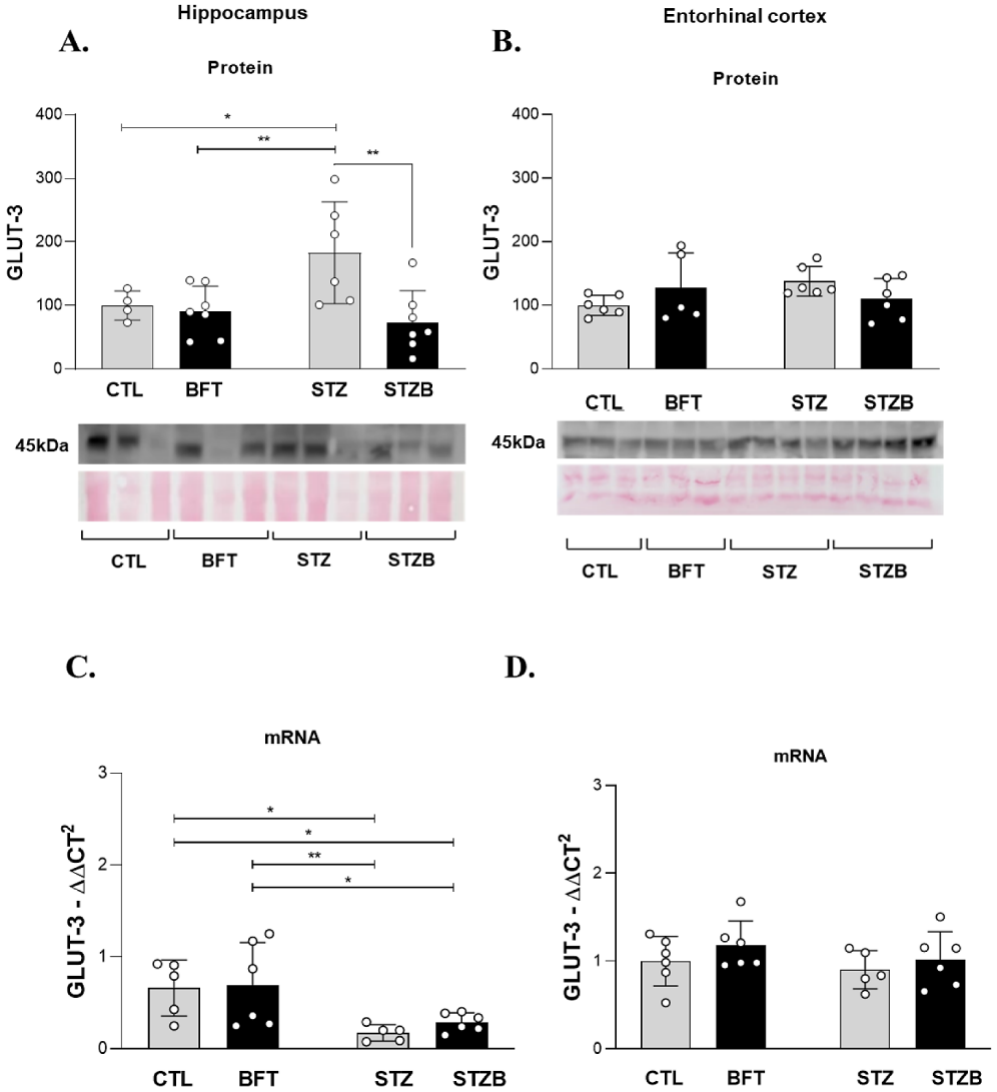
Effect of 7 day-BFT treatment in type 3 glucose transporters (GLUT3). (A) GLUT3 in the hippocampus detected by immunoblotting. (B) GLUT3 in the entorhinal cortex detected by immunoblotting. (C) mRNA levels of GLUT3 in the hippocampus. (D) mRNA levels of GLUT3 in the entorhinal cortex. Results are presented as mean ± SD (*n* = 4–7). *p < 0.05; **p < 0,0.1.

Evidence suggests that vascular and nonvascular glucose transporters (GLUTs) are altered in the AD brain, which could justify, at least in part, the glucose hypometabolism observed in AD patients.^56^ Therefore, we evaluated the levels of GLUT1 (vascular, 55 kDa) located in the brain barrier and GLUT3, most prevalent in neurons. We observed changes in GLUT1 levels and GLUT3 levels and expression only in the hippocampus. GLUT1 levels were 38.5% (F(1.19) = 3.999, *p* = 0.03) higher in STZ animals compared to BFT (Figure 8A). The STZB group had a decrease of 60.15% (F(1.19) = 3.999, *p* = 0.004), 35.7% (F(1.19) = 3.999, *p* = 0.03) and 74.15% (F(1.19) = 3.999, *p* = 0.0003) in the density of this transporter compared to CTL, BFT and STZ groups, respectively (Figure 8A). GLUT3 was 83.03% (F(1.20) = 5.024, *p* = 0.02) and 91.56% (F(1.20) = 5.024, *p* = 0.006) higher for STZ animals compared to CTL and BFT (Figure 9A). BFT treatment decreased 198.94% of GLUT3 levels for the STZB group compared to STZ (F(1.20) = 5.024, *p* = 0.001, Figure 9A). GLUT3 expression was lower for STZ (74.24%) and STZB (57.57%) compared to controls (F(1.18) = 0.111, *p* < 0.05, Figure 9C).

Our results show that treatment with BFT over a short period (7 days) impacts GLUTs levels. Decreases in GLUT1 and GLUT3 have been observed in brains affected by AD^56^ and this decrease is associated with decreased O-GlcNAcylation and abnormal hyperphosphorylation of tau in the brain affected by AD^66^ suggesting that the decrease may contribute to the pathology tau and neurodegeneration in AD. Deng et al., (2009) and Biswas et al., (2018) observed that GLUT1 and GLUT3 levels were also decreased in the brains of rats injected with STZ icv, suggesting that this decrease may be a result of dysfunctional insulin signaling caused by STZ.^66,67^ In our findings, STZ did not affect GLUT1; however, we evaluated the hippocampus and entorhinal cortex homogenate rather than the whole brain. On the other hand, neuronal transporters (GLUT3) were more sensitive to STZ damage. According to the literature,^66−68^ we observed a decrease in GLUT3 expression in the hippocampus. However, GLUT3 levels were higher in STZ animals, suggesting a lower degradation of these transporters possibly as a compensatory mechanism of STZ to the metabolic changes normalized by BFT treatment.

### STZ and Benfotiamine Alter Proteins Related to the Insulin Signaling Pathway in the Hippocampus and Entorhinal Cortex

Impaired insulin signaling can also result in deficits in glucose transport and metabolism.^69^ Despite many brain areas being insulin-independent for glucose uptake, compromises in the insulin signaling through the PI3K pathway decrease the expression of the hypoxia-inducible factor 1-α (HIF-1α), associated with a lower expression of GLUT1 and GLUT3^66^. Mullins et al. (2017) observed a positive correlation between GLUT1 and insulin signaling proteins, including insulin transporter substrate (IRS-1).^69^

The high concentration of insulin receptors in brain areas related to memory and learning suggests an essential role of this hormone in such a process.^70^ The binding of insulin to its receptor (IR) activates two main pathways: phosphoinositide 3-kinases (PI3K) protein kinase B (Akt) and mitogen-activated protein kinase (MAPK). To evaluate if BFT supplementation improves cognition through the insulin signaling pathway, we assessed the effect of the treatment on insulin receptors (IR) and insulin substrate receptor (IRS-1) phosphorylation.

**Figure 10:**
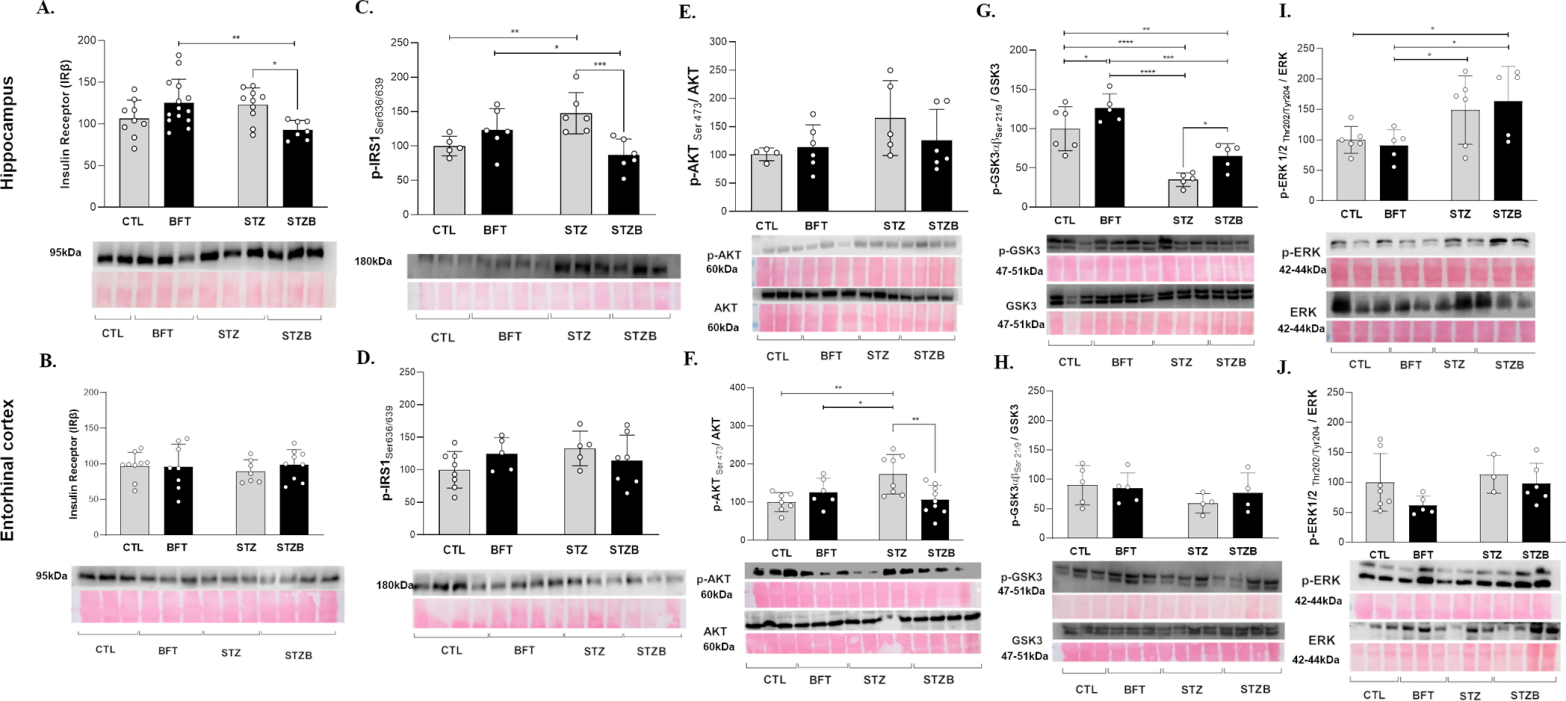
Effect of 7-day-BFT treatment in the protein levels of the insulin signaling pathway detected by immunoblotting. (A) Insulin receptor β subunit (IRβ) in the hippocampus and (B) in the entorhinal cortex. (C) p-IRS1_Ser636/639_ in the hippocampus and (D) in the entorhinal cortex. (E) pAKT_Ser473_/AKT in the hippocampus and (F) in the entorhinal cortex. (G) pGSK3_Ser21/9_/GSK3 in the hippocampus and (H) in the entorhinal cortex. (I) pERK1/2_Trh202/Tyr204_/ERK in the hippocampus and (J) in the entorhinal cortex. Results are presented as mean ± SD (*n* = 3–14). *p < 0.05; **p < 0,.1; ***p < 0.001; ****p < 0.0001.

In the hippocampus, the IRβ levels decreased by 32.61% and 30.28% in the STZB group compared to BFT (F(1.36) = 11.05, *p* = 0.003) and STZ (F(1.36) = 11.05, *p* = 0.01) (Figure 10A). IRS-1_Ser636/639_ phosphorylation increased by 47.61% for STZ animals (F(1.19) = 14.90, *p* = 0.007) compared to CTL in the hippocampus (Figure 10C). We did not observe differences in the entorhinal cortex (Figure 10B and D). Increased levels of phosphorylation in specific sites of the IRS-1, such as Serine_616_ and Serine_636/639_, can indicate insulin resistance.^11^

We did not observe changes in Akt phosphorylation in the Serine_473_ (pAKT_Ser473_/AKT) in the hippocampus (Figure 10E), commonly related to insulin signaling. In the entorhinal cortex, Akt levels increased by 76.03% (F(1.26) = 10.13, *p* = 0.001) and 48.9% (F(1.26) = 10.13, *p* = 0.03) in the STZ group compared to CTL and BFT, respectively (Figure 10F). BFT treatment decreased 67.9% (F(1.26) = 10.13, *p* = 0.001) of Akt phosphorylation in the STZB group compared to STZ.

The ratio between glycogen Synthase Kinase 3 (GSK3α/β) phosphorylation and total (pGSK3_Ser21/9_/GSK3) was also evaluated. We observed an increase in the enzymatic activity of GSK3 (i.e., decreased phosphorylation) induced by STZ in the hippocampus (64.6%, F(1.17) = 0.043, *p* < 0.001) (Figure 10G). BFT treatment increased GSK3 phosphorylation in STZB animals (27.71%, F(1.17) = 0.043, *p* = 0.02) compared to STZ in the hippocampus, indicating a positive effect of the treatment. In the entorhinal cortex, we did not observe differences between groups (Figure 10H).

The increase in extracellular signal-regulated kinase (ERK1/2_Trh202/Tyr204_) phosphorylation was identified only in the hippocampus after STZ injection. We observed an increase of 49.1% (F(1.18) = 0.414, *p* = 0.02) and 58.1% (F(1.18) = 0.414, *p* = 0.04) in the phosphorylation levels of this protein for STZ animals compared to CTL and BFT (Figure 10I), respectively. BFT treatment also increased ERK phosphorylation in the STZB group (73.13%, F(1.18) = 0.414, *p* = 0.01) compared to BFT. In the entorhinal cortex, we did not observe differences between groups (Figure 10J).

We observed impairments in insulin signaling, predominantly in the hippocampus. Although our findings highlight the hippocampus as the main affected area, our previous studies show gradual deficits in the entorhinal cortex and hypothalamus.^28,29^ Insulin resistance is positively correlated to increased levels of IRS-1 phosphorylation in Serine_616_ and Serine_636/639_, leading to cognitive impairment and an increase in Aβ deposits.^11^ In addition, the activation of the PI3K-AKT pathway regulates the activity of GSK3. GSK3 is fundamental for the modulation of LTP and long-term depression (LTD).^71^ The increase in GSK3 activity by deficits in insulin signaling can lead to neurofibrillary tangles formation through Tau hyperphosphorylation and Aβ plaques buildup.^72^ The ERK protein has increased activity in AD, phosphorylating Tau and contributing to the formation of neurofibrillary tangles.^73^ As previously indicated, oxidative stress activates ERK signaling, favoring the apoptotic pathway.^74,75^

Our findings demonstrate that BFT treatment normalizes IRS-1 phosphorylation and inhibits GSK3 activity in the hippocampus in STZB animals. These changes can be associated with the positive performance of this group in the cognitive tests, reinforcing the modulatory effect of BFT in insulin signaling. Other studies also showed the influence of BFT on cognitive parameters.^22,24,28,60^ Nonetheless, our work is the first to demonstrate a short-term effect of BFT in the early stages of STZ-induced neurodegeneration.

### STZ Favors Apoptotic Pathways in the Hippocampus and Entorhinal Cortex and Benfotiamine Treatment Decreases BAX Levels in the Hippocampus

**Figure 11:**
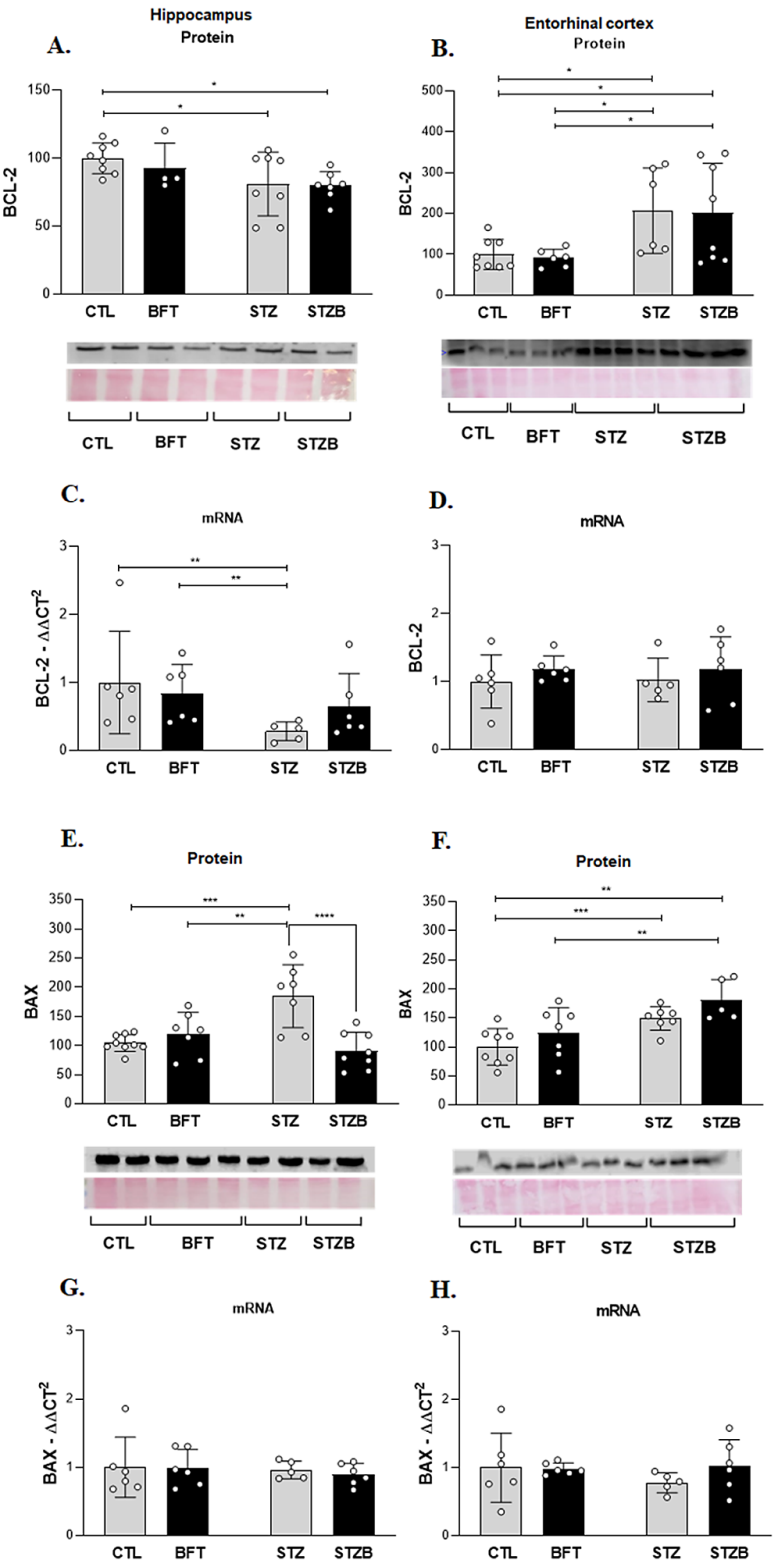
Effect of 7-day-BFT treatment in the protein levels of the apoptotic pathway detected by immunoblotting. (A) Bcl-2 levels and (B) Bcl-2 mRNA in the hippocampus. (C) Bcl-2 levels and (D) mRNA in the entorhinal cortex. (E) BAX levels and (F) BAX mRNA in the hippocampus. (G) BAX levels and (H) mRNA in the entorhinal cortex. Results are presented as mean ± SD (*n* = 5–6). *p < 0.05; **p < 0.01; ***p < 0.001.

BDNF and the insulin signaling pathway regulate pro- and antiapoptotic proteins. Proteins related to apoptosis were affected by STZ and BFT. We evaluated the levels and expression of antiapoptotic B-cell lymphoma 2 (Bcl-2) and pro-apoptotic Bcl-2-associated X protein (BAX). In the hippocampus, STZ and STZB groups showed a decrease of 20.9% (F(1.23) = 0.206, *p* = 0.03) and 21.3% (F(1.23) = 0.206, *p* = 0.02) in Bcl-2 levels compared to CTL (Figure 11A). The STZ group also presented a decrease of 72% in Bcl-2 expression (F(1.19) =1.492, *p* = 0.03) compared to CTL (Figure 11C). In the entorhinal cortex, STZ and STZB groups had an increase of approximately 107% (F(1.24) = 0.003, *p* = 0.02) in Bcl-2 levels compared to CTL and BFT (Figure 11B).

BAX levels increased by 80.4% (F(1.27) = 17.630, *p* = 0.0001) and 65.5% (F(1.27) = 17.630, *p* = 0.002) in the hippocampus of the STZ group compared to CTL and BFT, respectively (Figure 11E). BFT normalized BAX levels in the STZB group. There was a 93.5% (F(1.27) = 17.630, *p* < 0.0001) reduction in BAX levels for this group compared to STZ. In the entorhinal cortex, BAX levels increase by 49.2% (F(1.23) = 0.080, *p* = 0.009) and 80.7% (F(1.23) = 0.080, *p* = 0.003) for STZ and STZB compared to CTL, respectively. BFT treatment had no effect on BAX levels in this brain region (Figure 11G).

Insulin also plays a key role in MAPK-mediated brain antiapoptotic signaling.^76^ Despite observing increased ERK levels in the STZ groups, which may result from the increase in oxidative stress caused by STZ, our results indicate a protective effect of BFT, especially in the hippocampus where BAX levels are normalized during treatment, favoring the survival of neuronal cells by mechanisms that still need to be elucidated.

## Conclusion

Overall, we demonstrated that short-term BFT supplementation (7 days) reversed cognitive deficits in rats subjected to STZ-icv. Cognitive improvements may be associated with BFT’s ability to modulate glucose transporters, insulin signaling and apoptotic pathways. Therefore, BFT can be considered a promising cost-effective adjuvant intervention with positive outcomes in the initial treatment of Alzheimer’s-like disease.

## Limitations

There are several important limitations to our study that need to be considered. We did not evaluate blood or brain levels of benfotiamine metabolites, so we cannot determine whether the observed effects are due to a direct effect of BFT on the brain or whether they are reflective of a peripheral effect of the treatment. Furthermore, Alzheimer’s disease is a complex and multifactorial disorder, with several pathobiological subtypes that manifest in different cognitive ways. The STZ icv model partially reproduces the sporadic form of Alzheimer’s Disease, but has limitations in fully replicating its complexity or natural course. Therefore, regions close to the intracerebroventricular injection may present more exacerbated changes. We understand the importance of carrying out more comprehensive experiments, considering other changes characteristic of Alzheimer’s Disease, such as oxidative stress and neuroinflammation, to obtain a more detailed understanding of the changes involved.

## Methods

### Animals

Male Wistar rats (300–350g) were obtained from the Animal Facility of the Institute of Biomedical Sciences (ICB, Animal Facility Network at the University of Sao Paulo – USP). Animals were kept under controlled laboratory temperature conditions (23 °C) with a light/dark cycle (12h light/12h dark). Water and food were available *ad libitum*. All experimental procedures were conducted according to the Brazilian Law No. 11794 (October 08, 2008) and approved by the National Council for the Control of Animal Experimentation (CONCEA) and the Ethics Committee for Animal Use (CEUA – ICB, USP) (protocol n° 42960706/21 and 23/2016).

### Intracerebroventricular Injection of Streptozotocin (STZ)

The surgical procedure was performed in accordance with earlier laboratory studies, with minor adjustments.^28,29,44,51^ Thirty minutes before the surgery, the animals received subcutaneous methadone (2 mg/kg). Subsequently, animals were anesthetized with ketamine (100 mg/kg intraperitoneal) and xylazine (10 mg/kg intraperitoneal) and then placed in the stereotaxic apparatus (Kopf Instruments, Tujunga, CA, USA). They received a local subcutaneous injection of lidocaine (200 μL) before midline sagittal incision and skull display. According to the coordinates described in the literature,^77^ two small bilateral holes were made in the skull with a spherical drill for dental use. Animals received either STZ (2 mg/kg, Sigma-Aldrich, St. Louis, MO, United States) or vehicle (citrate buffer 0.05 mol/L, pH 4.5) injection in the lateral ventricles with glass micropipettes. Following the surgery, animals received a single dose of ketoprofen (5 mg/kg) and 5 daily doses of quinolone 2.5% (10 mg/kg).

### Benfotiamine Supplementation

**Figure 12:**
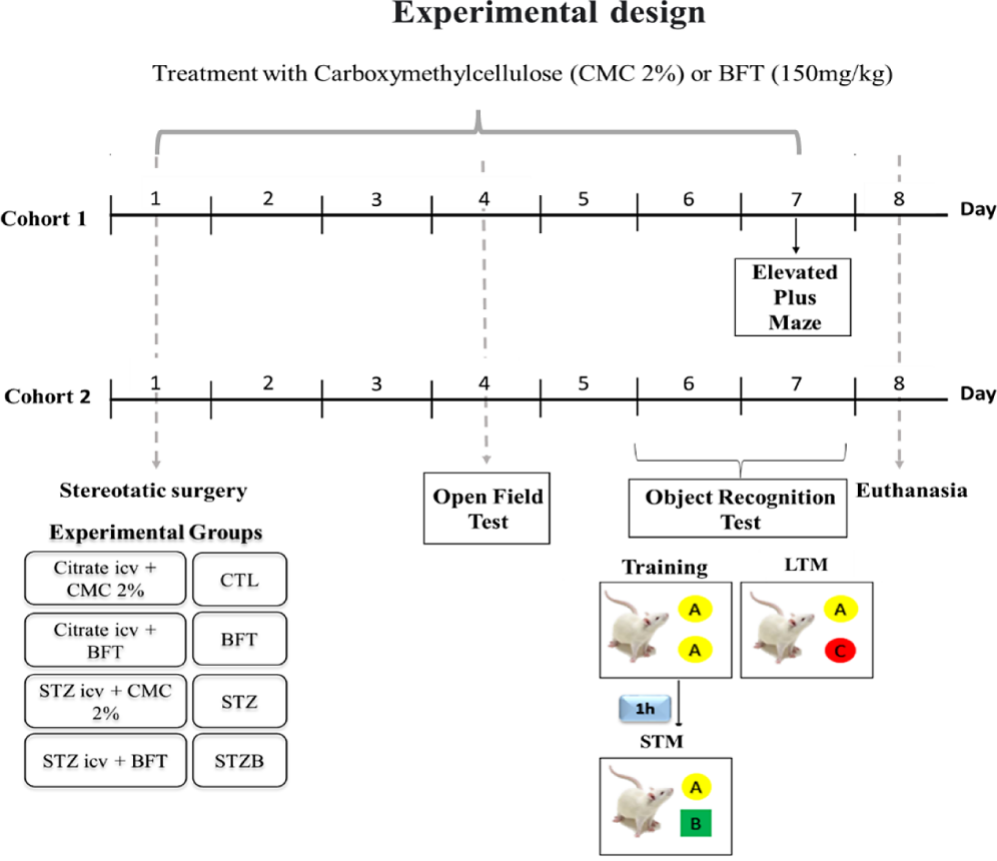
Experimental design. CMC = carboxymethylcellulose, BFT = benfotiamine, CTL = Control, STZ = streptozotocin, STZB = streptozotocin animals treated with benfotiamine, STM= short-term memory, LTM= long-term memory.

After surgery, animals received a daily oral dose of 150 mg/kg BFT (TCI Chemicals, Tokyo, Japan) or 2% Carboxymethylcellulose (CMC), used as a vehicle, for 7 days by gavage, as represented in Figure 12. BFT dose was based on previous works.^28,29^ BFT dilution in the vehicle was adjusted so that the total volume administered was equivalent to 1 mL/100 g, according to the animals’ stomach capacity and to avoid backflow. The animals constituted the groups: citrate icv treated with CMC (CTL); citrate icv treated with BFT (BFT); STZ icv treated with CMC (STZ); STZ icv treated with BFT (STZB). On the eighth day, animals were euthanized by decapitation after narcosis by isoflurane (Biochimico Institute, Itatiaia, RJ, Brazil). The hippocampus and entorhinal cortex were collected for immunoblotting and qPCR analysis.

### Open Field Test

The open field test (OF) was performed on the fourth day of treatment during the habituation phase of the object recognition test described below. The animals were placed in a circular open field arena divided into 12 zones for 10 min (acrylic; diameter 80 cm x height 50 cm). The OF test is commonly used to assess the anxious-like and exploratory behavior of rodents in a nonfamiliar environment. The parameters analyzed in the first 5 min of the test were: rearing, zone crossing, grooming, fecal boli count, total time in the center and time without exploration. The arena was cleaned with ethanol 5% to remove olfactory residues.

### Elevated Plus Maze

The elevated plus maze (EPM) is used to assess the anxious-like behavior of rodents. The test is based on the natural tendency of rodents to explore new environments and avoid unprotected, light and elevated places (open arms). The apparatus is elevated and composed of two close arms perpendicular opposed to two open arms. The animals were placed in the experimentation room for 30 min before the test. They were placed in the center of the maze with their head facing the open arm. Animals explored the apparatus for 5 min in a single section on the seventh day of treatment. The parameters analyzed were open arm entries (number of entries in the open arms/(number of entries in the open arms + number of entries in the closed arms) x 100), time spent in the open arms (time in the open arm/(time in the open arm + time in the closed arm) x 100), head dipping (exploratory movements of head and shoulder below the open arm surface), fecal boli count, and time in the center. The maze was cleaned with 5% ethanol in between animals.

### Object Recognition Test

The animals were submitted to the object recognition test, which involves the phases: habituation, training, and evaluation of the short- (STM) and long-term (LTM) memories (Figure 11). In the habituation phase, the animals were exposed to three daily sessions of 10 min with 1 h of interval between sessions in the OF arena for 2 days. In the training session (day 6), the animals were exposed to two identical objects (FO and FO’) for 10 min. Only animals that explored the objects for at least 30 s were included in the test phases. One hour and 24 h after the training session, STM and LTM were assessed, respectively. In the test phase, the animals were exposed to the FO and a new object (NO) for 5 min. In between animals, the arena was cleaned with 5% ethanol. The recognition index (RI) and discrimination ratio (DR) were calculated by
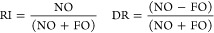


where NO is the time spent exploring the new object and FO is the time spent exploring the familiar object. The recognition task depends on the natural preference of animals in exploring the new object. RI > 0.5 indicates a preference for exploring the NO. DR represents the sensitivity of the recognition memory. Animals that do not show a preference for exploring the new are considered incapable of discriminating between the NO and the FO. Therefore, presenting a low recognition sensitivity (DR < 0).^78^

### Assessment of Mitochondrial Activity

Mice neuroblastoma neuro-2a cell line (ATCC, Richmond, VA, USA) were cultured in Dulbecco’s Modified Eagle Medium (DMEM, Gibco, NY, USA) supplemented with 1% penicillin–streptomycin and 10% FBS (FBS, Gibco, USA). Cells were maintained in 75 cm^2^ culture flasks at 37 °C and 5% CO_2_. After 48h of incubation, cells were treated with 5 mM STZ for 5 h before a 24 h treatment with 50 μM BFT.^27,51^ Oxygen consumption rates (OCR) of intact neuro-2a (2 × 10^6^ cells) were measured in a high-resolution Oxygraph (OROBOROS, Oxygraph-2k, Innsbruck, AU) in the presence of DMEM (10% FBS) medium. Subsequently, oligomycin A (F_o_-F_1_ ATP synthase inhibitor) and carbonylcyanide-p-trifluoromethoxyphenylhydrazone (FCCP, a potent ionophore used as uncoupler of mitochondrial oxidative phosphorylation, OxPhos) were sequentially titrated to reach optimum concentrations. To verify residual respiration, a mitochondrial complex III inhibitor, 5 μM Antimycin A, was added. ORC coupled to ATP production during OxPhos was calculated by the equation: OCR_ATP_ (pmol O_2_/min) = OCR_Routine_ (pmol O_2_/min) – OCR _Leak Respiration_ (pmol O_2_/min).^79^ Data were recorded and treated by using DatLab 8 software.

### Immunoblotting

Protein analysis was performed by immunoblotting as previously described.^28,29,44,51^ Briefly, brain regions were homogenized in an extraction buffer. Protein concentration was assessed by the Bradford method (Bio-Rad; Hercules, CA, USA). Samples containing 20–30 μg of protein were submitted to SDS-PAGE electrophoresis and transferred to nitrocellulose membranes (diameter 0.45 μm). Membranes were blocked with albumin solution (BSA 3%) for 1h and incubated with the primary antibodies listed in Table 1.

**Table 1:** Antibodies Used in the Immunoblotting

antibody	manufacture	code	species	clone
IRb	Santa Cruz	Sc-711	rabbit	polyclonal
pIRS*-*1_SER636/639_	Cell Signaling	2388	rabbit	polyclonal
AKT1/2/3	Santa Cruz	Sc-8312	rabbit	polyclonal
p-AKT1/2/3_SER__473_	Santa Cruz	Sc7985-r	rabbit	polyclonal
GSK3α*/*β	Santa Cruz	Sc -56 913	mice	monoclonal
p-GSK3α/β _SER21/9_	Cell Signaling	#9331	rabbit	polyclonal
ERK1/2	Santa Cruz	Sc -135 900	mice	monoclonal
P-ERK1/2_THR199/TYR202_	Cell Signaling	#1901	rabbit	polyclonal
BCL-2	Cell Signaling	#2876	rabbit	polyclonal
BAX	Cell Signaling	#2772	rabbit	polyclonal
GLUT-1	Abcam	ab652	rabbit	polyclonal
GLUT-3	Abcam	ab41525	rabbit	polyclonal
THTR-1	Santa Cruz	100649	mice	monoclonal

Following the incubation with the peroxidase-conjugated secondary antibody conjugated, the specific binding was revealed using chemiluminescence from luminol and p-coumaric acid (Sigma-Aldrich, USA). The optical density of the immunoreactivity of the band was captured in a C–Digit scanner (Li-Cor Inc., Lincoln, NE, USA) and analyzed by the ImageJ software (Fiji image analysis package - https://imagej.net/Fiji). Samples were normalized by Ponceau and results were presented as a percentage of the average of controls.

### qPCR

Tissues were homogenized in TRIzol (Invitrogen, Carlsbad, CA, USA). Total RNA was isolated with ReliaPrep RNA Tissue System Miniprep (Promega, Madison, WI, USA), and cDNA was synthesized from 1 μg of RNA with M-MLV reverse transcriptase, 50/50 mixture of oligo(dT), random primers, and RNase H (#M3681; Promega) following the manufacture instructions. The primer sequences used are listed in Table 2. qPCR was carried out with SYBR Green Real-Time Selected Master Mix (Applied Biosystems, CA, USA) and the optimal annealing temperature for PCR was 60 °C. Amplification and detection of PCR products were performed with ABI prism 7500 real Time-PCR System (Applied Biosystems). Gene expression was calculated by the 2^–ΔΔCt^ method using GAPDH and B2M as normalizing genes.

**Table 2:** Primers Used in the qPCR

gene	forward	melting temperature	reverse	melting temperature
THTR1	GATGCTCCTACGTACTGCCC	68	TGAAGACCTGTCTCTCGGTCA	67
mTPPTR	CAGCGCACTTTGTATGTGGT	64	AGGTTCCCCTGTTTGCTTTCCG	68
GLUT-1	ATGGGGACAGCGAAGGTGAC	67	TGGTTTAGCGTGCCAAATGC	65
GLUT-3	ATGGGGACAGCGAAGGTGAC	64	TGGTTTAGCGTGCCAAATGC	60
BDNF	ATGTTCCACCAGGTGAGAAG	64	GCCTTCATGCAACCGAAGTA	64
TRKβ	CTCCAACCTCAGACCACCAC	64	GCAGCACTTCCTGGGATAGG	64
Bax	CGGCAGTGATGGACGGG	63	TCGATCCTGGATGAAACCCTG	64
Bcl-2	TGCTAAGTTGCGAGTCCTGG	64	CACGTTTCTTGACCTGGGGC	64
GAPDH	CTCCCACTCCTTCCACCTTCG	64	CCACCACCCTGTTGCTGTAG	64
B2M	AATGTGAGGCGGGTGGAACTG	66	CATGGCTCGCTCGGTGACC	64

### Statistical Analysis

Data normality was assessed by Kolmogorov–Smirnov. For group comparison, normal data were analyzed with analysis of variance (ANOVA) two ways with Fisher LSD as post hoc test. Kruskal–Wallis was used for nonparametric data. The significant level adopted was 95%. Statistical analysis was performed in the GraphPad Prism. 8.0 software.

## Supporting Information Available

Effect of 7-day-BFT treatment in the motor function of rats in 5 min of the open field test and the table containing the two-way ANOVA statistical analysis (PDF)
