# Supplementary material for: Exploring the Therapeutic Potential of Benfotiamine in a Sporadic Alzheimer’s-Like Disease Rat Model: Insights into Insulin Signaling and Cognitive function
Source: ACS Chem Neurosci. 2024 Jul 15;15(16):2982–94. doi: 10.1021/acschemneuro.4c00113 (PMC11342302; doi:10.1021/acschemneuro.4c00113)
Supplement: Supplementary file 1 — cn4c00113_si_002.pdf [file cn4c00113_si_002.pdf]

Supporting information:

"Exploring the therapeutic potential of Benfotiamine in a sporadic Alzheimer's-like disease rat model: Insights into insulin signaling and cognitive function"

Camila, A. E. F. Cardinali [1a*], Yandara, A. Martins [1a], Ruan, C. M. Moraes [1,2], Andressa, P. Costa [1], Mayke B. Alencar [3], Ariel, M. Silber [3], Andrea S. Torrão [1]

[1]Departamento de Fisiologia e Biofisica, Instituto de Ciencias Biomedicas, Universidade de Sao Paulo, 05508-000, Sao Paulo, Brazil.

[2]Department of Psychiatry & Behavioral Neurosciences, The University of Alabama at Birmingham, 35294, Alabama, United States.

[3]Laboratory of Biochemistry of Tryps–LaBTryps, Departamento de Parasitologia, Instituto de Ciencias Biomedicas, Universidade de Sao Paulo, 05508-000, Sao Paulo, Brazil.

[a] Camila, A. E. F. Cardinali and Yandara, A. Martins have contributed equally to this study.

***Corresponding author:**

Camila Aparecida Errerias Fernandes Cardinali, Ph.D. student
Departamento de Fisiologia e Biofísica, Universidade de Sao Paulo
Av Professor Lineu Prestes 2415
05508-000 Sao Paulo, Brazil
Tel: +55 44 998889591
E-mail: camila.cardinali@icb.usp.br

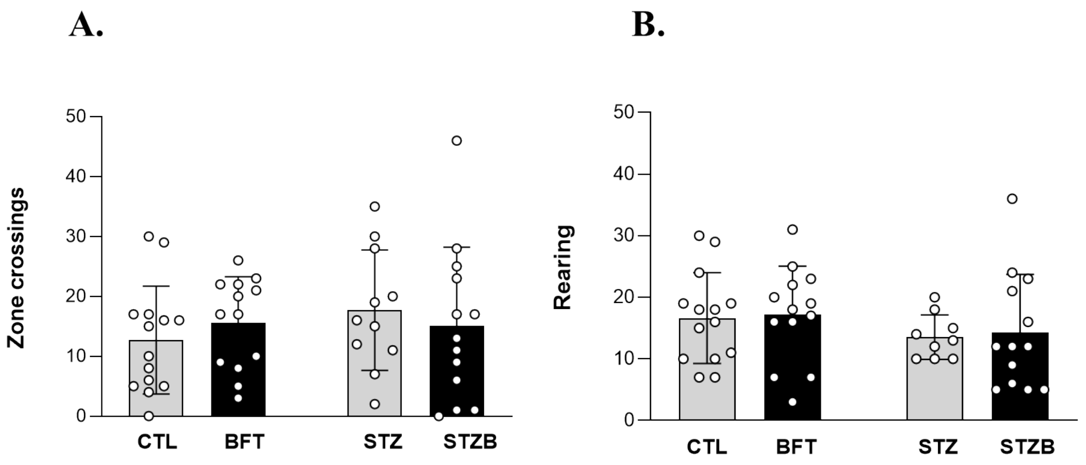

Figure 1S. Effect of 7-day-BFT treatment in the motor function of rats in 5 minutes of the open field test. **(A)** Zone crossings. **(B)** Rearing. Results are presented as mean±SD (n= 9-14).

Table 1S. Two-way ANOVA statistical analysis. Abbreviations are indicated in the text of the paper.

**Parameter**	**Figure**	**ANOVA table**	**DF**	**F(DFn, DFd)**	**P value**
Fecal boli count	Figure 1A	Interaction	1	F(1.47)=0.741	P=0.393
Grooming	Figure 1B	Interaction	1	F(1.41)=0.071	P=0.792
Time in the center	Figure 1C	Interaction	1	F(1.45)=1.099	P=0.300
Open arm entries	Figure 2A	Interaction	1	F(1.28)=0.037	P=0.847
Time on open arms	Figure 2B	Interaction	1	F(1.28)=0.142	P=0.708
Fecal boli count	Figure 2C	Interaction	1	F(1.28)=0.733	P=0.399
Head dippings	Figure 2D	Interaction	1	F(1.28)=0.003	P=0.956
Time in the center	Figure 2E	Interaction	1	F(1.23)=0.320	P=0.576
RI STM	Figure 3A	Interaction	1	F(1.41)=1.862	P=0.179
RI LTM	Figure 3B	Interaction	1	F(1.37)=8.730	P=0.005
DR STM	Figure 3C	Interaction	1	F(1.44)=7.896	P=0.007
DR LTM	Figure 3D	Interaction	1	F(1.39)=22.40	P<0.0001
Glun2b Hippocampus	Figure 4A	Interaction	1	F(1.17)=5.504	P=0.038
Glun2b Entorhinal cortex	Figure 4B	Interaction	1	F(1.19)=3.340	P=0.083
BDNF mRNA Hippocampus	Figure 5A	Interaction	1	F(1.19)=0.504	P=0.486
BDNF mRNA Entorhinal cortex	Figure 5B	Interaction	1	F(1.19)=0.080	P=0.780

TRKB mRNA Entorhinal cortex	Figure 5D	Interaction	1	F(1.19)=1.490	P=0.237
THTR-1 Hippocampus	Figure 7A	Interaction	1	F(1.31)=0.027	P=0.868
THTR-1 Entorhinal cortex	Figure 7B	Interaction	1	F(1.19)=9.290	P=0.006
THTR-1 mRNA Entorhinal cortex	Figure 7D	Interaction	1	F(1.20)=1.720	P=0.204
mTPPTR1 mRNA Hippocampus	Figure 7E	Interaction	1	F(1.18)=0.141	P=0.711
mTPPTR1mRNA Entorhinal cortex	Figure 7F	Interaction	1	F(1.19)=0.548	P=0.468
GLUT-1 Hippocampus	Figure 8A	Interaction	1	F(1.19)=3.999	P=0.060
GLUT-1 Entorhinal cortex	Figure 8B	Interaction	1	F(1.20)=0.521	P=0.478
GLUT-3 Hippocampus	Figure 9A	Interaction	1	F(1.20)=5.024	P=0.036
GLUT-3 Entorhinal cortex	Figure 9B	Interaction	1	F(1.19)=3.989	P=0.060
GLUT-3 mRNA Hippocampus	Figure 9C	Interaction	1	F(1.18)=0.111	P=0.741
GLUT-3 mRNA Entorhinal cortex	Figure 9D	Interaction	1	F(1.19)=0.066	P=0.799
IR Hippocampus	Figure 10A	Interaction	1	F(1.36)=11.50	P=0.002
IR Entorhinal cortex	Figure 10B	Interaction	1	F(1.29)=0.387	P=0.538
p-IRS1$_{ser636/639}$ Hippocampus	Figure 10C	Interaction	1	F(1.19)=14.90	P=0.001
p-IRS1$_{ser636/639}$ Entorhinal cortex	Figure 10D	Interaction	1	F(1.21)=2.908	P=0.102
pAKT$_{ser473}$/AKT Entorhinal cortex	Figure 10F	Interaction	1	F(1.26)=10.13	P=0.003
pGSK3$_{ser21/9}$/GSK3 Hippocampus	Figure 10G	Interaction	1	F(1.17)=0.043	P=0.837
pGSK3$_{ser21/9}$/GSK3 Entorhinal cortex	Figure 10H	Interaction	1	F(1.14)=0.714	P=0.412
pERK1/2$_{Trh202/Tyr204}$/ERK Hippocampus	Figure 10I	Interaction	1	F(1.18)=0.414	P=0.527

pERK1/2$_{Trh202/Tyr204}$/ERK Entorhinal cortex	Figure 10J	Interaction	1	$F(1.17)=0.480$	$P=0.497$
BCL-2 Hippocampus	Figure 11A	Interaction	1	$F(1.23)=0.206$	$P=0.653$
BCL-2 Entorhinal cortex	Figure 11B	Interaction	1	$F(1.24)=0.003$	$P=0.956$
BCL-2 mRNA Hippocampus	Figure 11C	Interaction	1	$F(1.19)=1.492$	$P=0.236$
BAX Hippocampus	Figure 11E	Interaction	1	$F(1.27)=17.63$	$P=0.0003$
BAX Entorhinal cortex	Figure 11F	Interaction	1	$F(1.23)=0.080$	$P=0.779$
